# PFOA and High Cholesterol: Basis for the Finding of a Probable Link

**DOI:** 10.1289/ehp.122-A338

**Published:** 2014-12-01

**Authors:** Kellyn S. Betts

**Affiliations:** Kellyn S. Betts writes about environmental contaminants, hazards, and technology for solving environmental problems for publications including *EHP* and *Environmental Science & Technology*.

The C8 Science Panel was created as part of a class action settlement to study the relationship between perfluorooctanoic acid (PFOA) and disease in the community surrounding DuPont’s Washington Works facility in West Virginia.^1^ The panel of three epidemiologists has judged there to be a “probable link”^2^ between exposure to PFOA and several health conditions: kidney and testicular cancers, pregnancy-induced hypertension, thyroid disease, ulcerative colitis, and high cholesterol.^3^ In this issue of *EHP*, panelist Kyle Steenland and colleague Andrea Winquist, both of Emory University, report part of the basis for the probable link with high cholesterol.^4^

**Figure:**
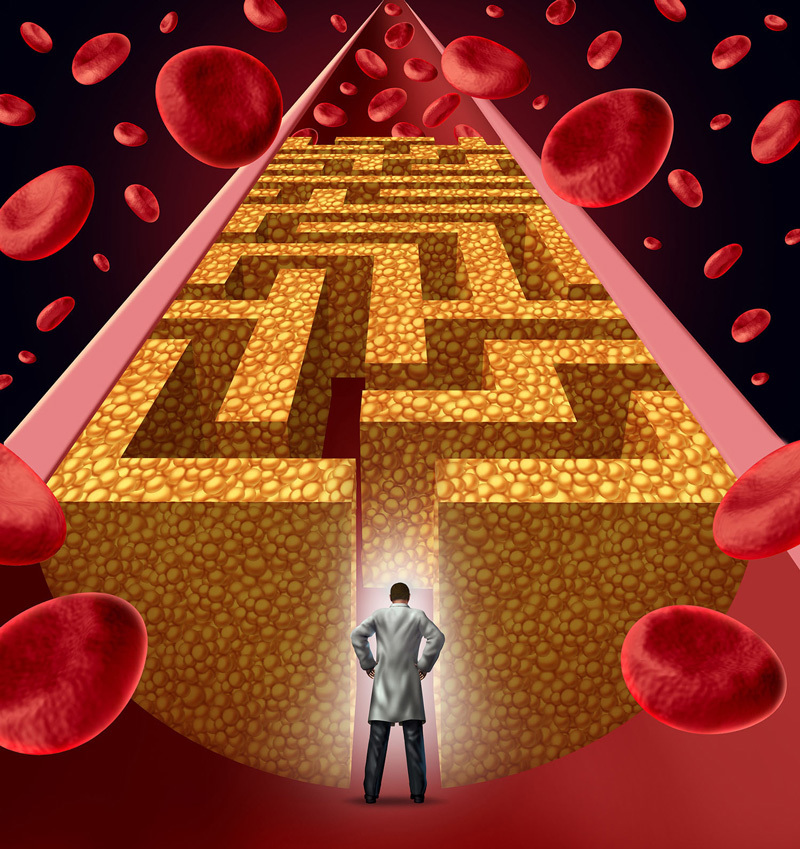
After assessing the existing scientific evidence and estimating community and worker exposures to PFOA, the C8 Science Panel concluded there is a “probable link” between PFOA exposure and high cholesterol among the population around DuPont’s Washington Works facility. © Lightspring/Shutterstock

Nearly all U.S. adults sampled in the nationally representative U.S. National Health and Nutrition Examination Survey (NHANES) between 1999 and 2008 had detectable levels of PFOA in their blood.^5^ The residents around the Washington Works facility, which produced PFOA for decades starting in the early 1950s, received unusually high exposures through contaminated drinking water; some also worked at the plant.^4^ At least 14 human studies, most of them cross-sectional in design, have linked PFOA exposure with heart disease risk factors (including higher levels of uric acid and homocysteine in serum) and higher serum cholesterol.^4^

The C8 Science Panel used several models to analyze data collected from 3,713 workers and 28,541 other members of the community.^6^ A fate and transport model with data on the plant’s PFOA emissions was used to estimate annual exposure of the community members through local air, surface water, and groundwater.^7^ To estimate yearly PFOA intakes, the panel combined modeled water concentration calculations with information about residential history, drinking water sources, and water consumption rates.^8^ They then used a pharmacokinetic model to approximate PFOA serum concentrations for community members. For workers, an occupational model generated job- and department-specific PFOA serum concentration estimates.^9^ For people who both lived in the community and worked at the plant, the analysis used whichever PFOA estimate was higher for any given year.

The panel analyzed associations between PFOA exposure and self-reported high cholesterol, coronary artery disease, and hypertension using Cox proportional hazard models. (No probable link was found with the latter two health conditions.^4^) They used various exposure metrics in the models to account for different potential biological mechanisms, says Winquist. The models also controlled for potential confounders including birth year, because the patterns of exposure changed over time.

The serum predictions from the exposure model correlated quite well with the levels actually measured in the worker and community populations, which “bodes well for their accuracy,” says Steenland.

The exposure calculations did not factor in food or indoor dust, which are believed to be important routes through which people in the general population are exposed to PFOA, points out Christopher Lau, a researcher of developmental toxicology at the U.S. Environmental Protection Agency (EPA) National Health and Environmental Effects Research Laboratory. But he agrees that the research’s focus is justified by the high levels of PFOA released into the local environment by the DuPont plant over its decades of operation. He adds that the facility’s emissions recently decreased by more than 97% in concordance with a consent agreement with the EPA.^10^

One weakness of the study was that it only included community members alive in 2005–2006, so it may have excluded people who developed coronary artery disease and died before that point. Although the workers did not need to be alive by 2006 in order to be included in the study, it was difficult to find proxies (typically family members) who could provide information about deceased workers.^4^

Steenland and his colleagues on the C8 Science Panel stress that class action lawsuits involving environmental contaminants have only rarely produced data evaluating whether the exposure in question actually caused health effects. They hope this settlement, with its research requirements, sets a precedent for future settlements.^11^
